# Prognostic Value of Intratumor Metabolic Heterogeneity Parameters on ^18^F-FDG PET/CT for Patients with Colorectal Cancer

**DOI:** 10.1155/2022/2586245

**Published:** 2022-01-30

**Authors:** Xin Liu, Kun Xiang, Guang-Yong Geng, Shi-Cun Wang, Ming Ni, Yi-Fan Zhang, Hai-Feng Pan, Wei-Fu Lv

**Affiliations:** ^1^Anhui Provincial Hospital, Cheeloo College of Medicine, Shandong University, Jinan, Shandong 250012, China; ^2^Department of Nuclear Medicine, The First Affiliated Hospital of USTC, Division of Life Sciences and Medicine, University of Science and Technology of China, Hefei, Anhui 230001, China; ^3^Department of Epidemiology and Biostatistics, School of Public Health, Anhui Medical University, 81 Meishan Road, Hefei, Anhui, China; ^4^Inflammation and Immune Mediated Diseases Laboratory of Anhui Province, 81 Meishan Road, Hefei, Anhui, China; ^5^Department of General Surgery, The Fourth Affiliated Hospital of Anhui Medical University, Hefei, Anhui 230001, China

## Abstract

**Purpose:**

Intratumor metabolic heterogeneity parameters on ^18^F-2-fluoro-2-deoxy-D-glucose (^18^F-FDG) positron emission tomography-computed tomography (PET-CT) have been proven to be predictors of the clinical prognosis of cancer patients. The study aimed to examine the correlation between ^18^F-FDG PET-CT-defined heterogeneity parameters and the prognostic significance in patients with colorectal cancer.

**Methods:**

The study included 188 patients with colorectal cancer who received surgery and ^18^F-FDG PET/CT examinations. Preoperative ^18^F-FDG PET/CT conventional and metabolic heterogeneity parameters were collected, including maximum, peak, and mean standardized uptake value (SUVmax, SUVpeak, and SUVmean), metabolic tumor volume (MTV), total lesion glycolysis (TLG), heterogeneity index-1 (HI-1) and heterogeneity index-2 (HI-2), and clinicopathological information. Correlations between these parameters and patient survival outcomes were inferred.

**Results:**

The associations between ^18^F-FDG PET/CT parameters and clinical outcomes were analyzed. Tumor thrombus (*P* < 0.001), tumor stage (*P*=0.001), MTV (*P*=0.003), HI-1 (*P*=0.032), and HI-2 (*P*=0.001) differed between the two groups with and without recurrence. Multivariate analysis showed that, in the radical surgery group, HI-2 (HR = 1.10, 95% CI: 1.04–1.17, *P*=0.001), tumor stage (HR = 20.65, 95% CI: 4.81–88.62, *P* < 0.001), and regional lymph nodes status (HR = 0.16, 95% CI: 0.04–0.57, *P*=0.005) were independent variables significantly correlated with progression-free survival (PFS) and HI-2 (HR = 1.16, 95% CI: 1.07–1.26, *P* < 0.001) was an independent variable affecting overall survival (OS). In the palliative surgery group, HI-2 (HR = 1.03, 95% CI: 1.01–1.06, *P*=0.020) was an independent variable affecting PFS, and all the parameters were not statistically significant for OS.

**Conclusion:**

HI-2, tumor stage, and regional lymph nodes status might predict the outcomes of colorectal cancer more effectively than other ^18^F-FDG PET/CT defined parameters.

## 1. Introduction

Colorectal cancer is one of the most common malignancies worldwide, with a dismal prognosis. In 2018, there were approximately 1.8 million newly reported cases of colorectal cancer and about 861,000 related deaths [1]. Nearly 20% of patients are at an advanced stage at the time of diagnosis and unable to receive curative surgery due to extensive metastatic properties [2]. For nonmetastasized colorectal cancer (T_1-4_N_0-2_M_0_), radical surgical resection is the most effective treatment, but for locally advanced middle and lower rectal cancer with _C_T_3-4_ and/or N+, neoadjuvant chemoradiotherapy or neoadjuvant chemotherapy is recommended before surgical treatment. In addition, for late period colorectal cancer, surgery, radiotherapy, chemotherapy, or targeted therapy can be chosen according to the patient's condition [3, 4]. Despite considerable advances in colorectal cancer treatment, the 5-year survival rate of colorectal cancer patients treated with surgery is still less than 50% [1]. The death of colorectal cancer is mainly related to distant metastasis at the time of diagnosis or after the cancer-free period [5]. At present, the tumor-node-metastasis (TNM) staging system of the American Joint Committee on Cancer (AJCC) is the most common predictive model for colorectal cancer. In addition, the predictive effect of biological markers and molecular markers, including CEA, the RAS gene, BRAF, and HER-2, also plays a critical role in clinical practice in recent years.

^18^F-2-fluoro-2-deoxy-D-glucose (^18^F-FDG) positron emission tomography-computed tomography (PET/CT) is an imaging method to measure and quantify the metabolic avidity of cancer tissues, thereby acting as a proxy for essential cell activity and viability [6]. The ^18^F-FDG PET/CT has been proven to be effective for diagnosing, staging, prognosis prediction, and treatment response assessment in numerous cancers. Conventional metabolic parameters, including metabolic tumor volume (MTV), total lesion glycolysis (TLG), and maximum and peak standardized uptake value (SUVmax/SUVpeak), have been proven to be effective for predicting the survival outcomes of cancer patients [7–9]. The concept of intratumor metabolic heterogeneity poses a challenge to patients' molecular stratification and treatment guidelines using a single tumor tissue sample [10]. In recent years, the ideas of ^18^F-FDG PET/CT intratumor metabolic heterogeneity parameters, such as coefficient of variance, which is calculated through SUVmean divided by the standard deviation, and the slope of linear regression, which is calculated through linear regressions of MTVs according to different SUV thresholds, have been proven to reflect the characteristics of intratumor heterogeneity to some extent and to help predict prognosis in some solid tumors [11–15]. However, the intratumor metabolic heterogeneity prognostic value in colorectal cancer has not been studied.

A retrospective study was conducted to assess the predictive value of intratumor metabolic heterogeneity parameters of ^18^F-FDG PET/CT for patients with colorectal cancer.

## 2. Materials and Methods

### 2.1. Study Subjects

**Figure 1:**
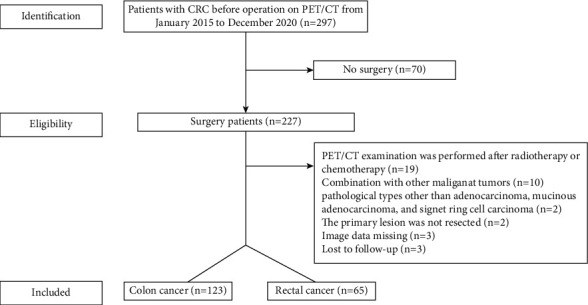
Flowchart of patient screening.

This study reviewed the preoperation ^18^F-FDG PET/CT of 297 consecutive patients with pathologically proven colorectal cancer in the First Affiliated Hospital of USTC from January 2015 to December 2020. Of these, we excluded 70 patients who did not receive surgery, 19 patients who received other treatments before PET/CT, 10 patients with secondary tumor, 2 patients with a pathological type of squamous cell carcinoma, 2 patients with unresectable primary mass, 3 patients with incomplete image data, and 3 patients who were lost to follow-up. Ultimately, this study enrolled 188 patients (Figure 1). The inclusion criteria were as follows: (1) colorectal cancer was pathologically confirmed as adenocarcinoma, mucinous adenocarcinoma, or signet ring cell carcinoma; pathological tumor features were obtained from the surgical pathology report; (2) serum carcinoembryonic antigen (CEA) and carbohydrate antigen 19-9 (CA19-9) levels were detected within one week of PET/CT examination; (3) without any treatment before PET/CT examination, radical or palliative surgery was performed within two weeks after the examination; (4) the tumor tissue showed positive FDG metabolism. The exclusion criteria for the subjects were as follows: (1) colorectal cancer of other pathological types; (2) colorectal cancer accompanying a second primary malignant tumor. The research protocol was approved by the Medical Ethics Committee of the First Affiliated Hospital of USTC (2021-RE-002).

### 2.2. ^18^F-FDG PET/CT Examination

The scanning process was performed on the Siemens Biography Sensation 16 PET/CT imager (Siemens Medical Systems Group, Knoxville, Tennessee, USA) with a 4.0 mm full width at half maxima and 16.2 cm axis field width. Before the examination, the patients needed to be fasting for more than 6 hours. When the blood glucose level reached the normal range (<11.1 mmol/L), ^18^F-FDG (3.7–7.4 MBq/kg) was injected intravenously into the patients. Initially, a low-dose CT scan was performed from the middle part of the eye to above the upper femur. CT scan parameters were 120 kV, 100 mA, pitch 0.75, slice thickness 5 mm (automatic reconstruction 3 mm), interval 5 mm, and matrix size 512 × 512. Then, the PET scan was conducted in a three-dimensional model. According to the CT scanning field, 6-7 beds were generally collected, with a 2-minute collection time for each bed. Subsequently, attenuation correction of PET data was performed based on CT data, and the ordered subset maximum expected iteration method was used for reconstruction. Finally, PET images and CT images were automatically generated on the workstation.

### 2.3. Semiquantitative Analysis of Tumor PET/CT Images

**Figure 2:**
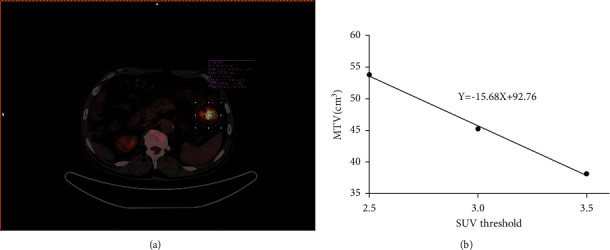
Process of measuring metabolic heterogeneity indices on ^18^F-FDG PET/CT. Fused PET/CT image showing an ^18^F-FDG avid tumor in the colon. A circle (pink) was drawn to include the whole tumor, and an isocontour volume of interest (VOI; pink) was automatically generated by using a 40% SUVmax cutoff (a). Heterogeneity index-1 was defined as the coefficient of variance, which was calculated as the SD of the SUV divided by SUVmean. Metabolic tumor volume (MTV) was assessed according to three thresholds (SUV 2.5, 3.0, and 3.5, resp.), and linear regression analysis was performed to find the slope. Heterogeneity index-2 was the negative form of the slope (b).

The primary colorectal cancer lesion location on the PET/CT images was determined by the Siemens Syngo Via workstation (Siemens Medical Systems Group, Knoxville, Tennessee, USA). The largest diameter of the tumor along the direction of the bowel was measured. The 40% SUVmax served as the threshold to establish the volume of interest (VOI) to measure the metabolic parameters and the volume parameters of the lesion, including SUVmax, SUVpeak, SUVmean, MTV, and TLG. In addition, HI-1 and HI-2 were calculated. HI-1 is the ratio of the standard deviation of SUV to SUVmean, also known as the variance coefficient [13]. HI-2 is the negative form of the linear regression slope of MTV calculated according to different SUV thresholds (2.5, 3.0, and 3.5) [11], calculated by a slight improvement of previous methods [14, 15] (Figure 2). The independent evaluation of the images was performed by two nuclear medicine doctors with over five-year working experience, who were required to be blinded to each other's opinion. Once disagreements emerged on the primary tumor location, the decision would be made by a superior doctor.

### 2.4. Patients Follow-Up

Patients with colorectal cancer who underwent surgery would go to the hospital for regular review. If the patients had received other treatments (radiotherapy, chemotherapy, etc.) after the surgery, the CEA was tested before each treatment, and the CEA should be reviewed regularly at least every 3-4 months in the first three years and every 4-6 months after that for patients without subsequent treatment. CT and MRI imaging were used for follow-up during the review period, and the Response Evaluation Criteria in Solid Tumors (RECIST) were used to confirm whether the disease was progressing. Recurrence was defined as recurrence or metastasis with a positive biopsy or clear clinical/radiological evidence after radical surgery. Progression-free survival (PFS) was defined as the time from surgery to disease recurrence, progression, or death. Overall survival (OS) was defined as the time from surgery to death.

### 2.5. Statistical Analysis

The Shapiro–Wilk test was used to test the normal distribution of each variable. Continuous data were represented as medians (interquartile ranges) or mean ± standard deviation (SD), while categorical data were represented as proportions. The patients were divided into two groups according to pathological characteristics, including T_1-2_/T_3-4_, regional lymph nodes (N)-/N+, distant metastasis (M)-/M+, tumor thrombus (TT)-/TT+, nerve invasion (NI)-/NI+, pathological types, differentiation degree, and the tumor location. The differences in PET parameters were compared between the groups. Patients who underwent radical surgery were divided into two groups according to tumor recurrence, and the differences in clinicopathological characteristics and PET parameters were compared. Chi-square test or Fisher's exact test, *t*-test, and Mann–Whitney *U* test were conducted for comparison between groups. According to radical and palliative treatment groups, Kaplan–Meier survival analysis with the log-rank test was conducted to obtain a survival curve, and univariate and multivariate Cox proportional hazards' regression was conducted to assess the associations between the parameters and PFS/OS. The optimal threshold of parameters was obtained using the receiver operating characteristic (ROC). Data analysis was performed on R software (version 4.0.3, University of Auckland, New Zealand).

## 3. Results

### 3.1. Demographics

Over the period between January 2015 and December 2020, 188 patients with colorectal cancer were selected as participants of the study, consisting of 114 males and 74 females, with an average age of 65 years old (range: 34–91). The collected information included the tumor location, the degree of differentiation, pathological types, tumor length, T stage, regional lymph nodes metastasis, distant metastasis, nerve invasion status, tumor thrombus status, tumor stage (AJCC), CEA, and CA19-9. The detailed information is shown in Table 1.

### 3.2. Differences in ^18^F-FDG PET/CT Parameters between Groups with Different Pathological Characteristics

The subjects were grouped according to T_1-2_/T_3-4_, N−/N+, M−/M+, TT−/TT+, NI−/NI+, adenocarcinoma/mucinous adenocarcinoma (MAC) and signet ring cell carcinoma (SRC), low-differentiation (LD)/mid-differentiation (MD) and high-differentiation (HD), and right colon/left colon. There were no significant differences between all groups in SUVmax, SUVmean, SUVpeak. Patients with T_3-4_, TT+, MAC and SRC, LD, and right colon had significantly higher MTV of primary tumor than those with T_1-2_, TT−, adenocarcinoma, MD and HD, and left colon (*P*=0.028, *P*=0.015, *P*=0.011, *P*=0.008, and *P*=0.022, resp.). TLG in the T_3-4_, right colon groups were higher than those in the T_1-2_, left colon groups (*P*=0.046; *P*=0.029). HI-1 differed in different pathological types (*P*=0.045), while HI-2 differed between all the groups except the N−/N+ group (*P*=0.002, *P*=0.046, *P*=0.024, *P*=0.045, *P*=0.011, *P*=0.006, and *P*=0.018, resp.) (detail in Supplementary Table 1).

### 3.3. Recurrence

All 188 patients underwent surgical treatment. For 59 patients (Stage IV), the primary lesion was resected, but the metastatic lesion was not resected. 129 patients (Stage I to Stage III) underwent radical surgery and achieved a tumor-free state after operation. Among the 129 patients, 29 patients had tumor recurrence during the follow-up. After comparing the parameters of the patients with and without recurrence, significant differences could be found in tumor thrombus (*P* < 0.001), tumor stage (*P*=0.001), MTV (*P*=0.003), HI-1 (*P*=0.032), and HI-2 (*P*=0.001) between the two groups (Table 2).

### 3.4. Univariate and Multivariate Analyses of PFS/OS

During follow-up, among the 129 patients (Stage I to Stage III), 33 of them had tumor recurrence or died in PFS analysis, and 8 of them died in OS analysis. The average PFS was 50.03 months (95% CI, 44.40–55.66), while the average OS was 64.32 months (95% CI, 61.09–67.55). Among the 59 patients (Stage IV), 40 of them had disease progression or died in PFS analysis, and 14 of them died in OS analysis. The average PFS was 17.12 months (95% CI, 13.68–20.56), while the average OS was 31.41 months (95% CI, 27.66–35.16).

**Figure 3:**
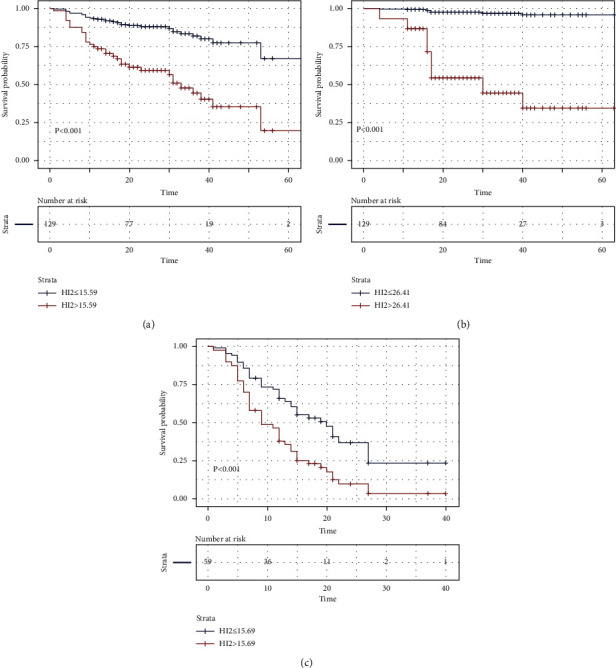
Kaplan–Meier curves for comparing prognosis according to HI-2 (a). Kaplan–Meier curves of PFS stratified by HI-2 (cutoff 15.59) in the radical treatment group (b). Kaplan–Meier curves for comparing OS according to HI-2 (cutoff 26.41) in the radical treatment group (c). In the palliative treatment group, patients with higher HI-2 (cutoff 15.69) had more severe prognosis.

In the radical surgery group, the univariate analysis demonstrated that regional lymph nodes status (HR = 2.54, 95% CI: 1.26–5.12, *P*=0.009), tumor thrombus status (HR = 3.85, 95% CI: 1.90–7.80, *P* < 0.001), tumor stage (HR = 3.67, 95% CI: 1.74–7.75, *P*=0.001), MTV (HR = 1.02, 95% CI: 1.01–1.04, *P*=0.012), and HI-2 (HR = 1.07, 95% CI: 1.04–1.10, *P* < 0.001) (Figure 3(a)) were significantly correlated with PFS. Multivariate analysis showed that HI-2 (HR = 1.10, 95% CI: 1.04–1.17, *P*=0.001), tumor stage (HR = 20.65, 95% CI: 4.81–88.62, *P* < 0.001), and regional lymph nodes status (HR = 0.16, 95% CI: 0.04–0.57, *P*=0.005) were independent variables which were significantly correlated with PFS. In univariate analysis, MTV (HR = 1.03, 95% CI: 1.00–1.06, *P*=0.016) and HI-2 (HR = 1.10, 95% CI: 1.05–1.15, *P* < 0.001) (Figure 3(b)) were associated with OS. In the multivariate analysis, HI-2 (HR = 1.16, 95% CI: 1.07–1.26, *P* < 0.001) was an independent variable affecting OS (Table 3).

In the palliative surgery group, the univariate analysis demonstrated that SUVpeak (HR = 1.07, 95% CI: 1.01–1.13, *P*=0.025), TLG (HR = 1.00, 95% CI: 0.99–1.00, *P*=0.012), and HI-2 (HR = 1.04, 95% CI: 1.02–1.07, *P*=0.001) (Figure 3(c)) were significantly related to PFS. In the multivariate analysis, except for HI-2 (HR = 1.03, 95% CI: 1.01–1.06, *P*=0.020), none of the variables were independent variables affecting PFS. In the univariate analysis for OS, all the parameters were not statistically significant (Table 4).

## 4. Discussion

To our knowledge, this is the first study to explore the predictive value of ^18^F-FDG PET/CT intratumoral HI in colorectal cancer patients. The current research indicates that the intratumoral HI-2 of ^18^F‐FDG uptake is an important prognostic factor for PFS and OS in patients with colorectal cancer.

Tumor heterogeneity refers to the fact that, during the growth process of tumor tissue, after multiple division and proliferation, its daughter cells show changes in molecular biology or genes, leading to differences in growth rate, invasion ability, susceptibility to drugs, and prognosis, which can be seen as one of the characteristics of the malignant tumor. Tumor heterogeneity is one of the obstacles to personalized medicine, which always leads to treatment failure [16]. Tumor heterogeneity may be related to disease progression, malignant behavior of the tumor, and patients' response to treatment [17]. The predictive value of tumor heterogeneity characterization draws extensive attention in the field of ^18^F-FDG PET/CT tumor imaging. ^18^F-FDG PET/CT imaging mainly uses conventional indicators such as SUV, MTV, and TLG and image-omics texture parameters (i.e., a series of parameters based on texture analysis) to quantify intratumoral heterogeneity.

The conventional metabolic parameters are commonly used in the clinic as useful prognostic factors for colorectal cancer patients. SUVmax and ^18^F-FDG volume parameters have been proven to predict the prognosis of patients with colorectal cancer [18–22]. Although the conventional parameters can reflect the heterogeneity characteristic of cancer to some extent, there are still certain limitations. SUV can only reflect activity at one point within the tumor rather than the overall metabolism of the tumor. MTV and TLG can make up for this shortcoming. These parameters can reflect the metabolic information in the entire tumor, which may be more accurate for tumor characterization than single-voxel measurements [23]. However, MTV and TLG cannot distinguish the heterogeneity of different regions within the tumor.

Although HI needs to be calculated compared to conventional ^18^F-FDG parameters, it can distinguish the metabolic differences in different tumor regions. In some studies which included both conventional and heterogeneous PET parameters, heterogeneous parameters played a more significant role in predicting the prognosis than the conventional parameters [13, 24]. This evidence underscores the importance of tumor heterogeneity parameters and their potential to predict clinical outcomes in cancer patients.

HI-1, as the ratio of the standard deviation of SUV to SUVmean, also known as the coefficient of variation, reflects the degree of variation of SUV. Lee et al. showed that high HI-1 was associated with epithelial ovarian cancer recurrence [13]. Chung et al. found that preoperative HI-1 was the only independent risk factor for cervical cancer recurrence [24]. However, we found that HI-1 was significantly higher in adenocarcinoma patients and patients without tumor recurrence in the radical surgery group but not associated with the survival prognosis of colorectal cancer. The reason may be related to the thresholds option. VOI may produce different SD and SUVmean under different SUV thresholds, and the optimal thresholds for VOI delineation differ under various cancer types. The results suggest that HI-1 calculated with 40%SUVmax as the threshold may not be applicable to predict heterogeneity of colorectal cancer.

HI-2, as the negative form of the linear regression slope of MTV calculated according to different SUV thresholds (2.5, 3.0, and 3.5), represents the MTV discrepancy under different SUV thresholds. According to a study based on the Chinese population, the HI-2 value was significantly related to the survival outcome of gastric cancer patients; higher HI-2 indicated poor prognosis [11]. Kwon et al. indicated that HI-2 was one of the independent predictors of overall survival in oral cavity cancer. Patients with higher HI-2 showed a worse prognosis than those with lower HI-2 [15]. A retrospective study suggested that higher HI-2 could be used to predict recurrence of pancreatic ductal adenocarcinoma [25]. Kim et al. found that survival of patients with more heterogeneous tumors (HI-2) was poorer than those with relatively homogeneous tumors [26]. In this study, it was found that HI-2 was significantly higher in the T3-4, TT+, M+, NI+, MAC, and SRC, LD, right colon groups and that HI-2 was associated with colorectal cancer prognosis. HI-2 highly reflects the intratumor heterogeneity, which has been demonstrated to correlate with treatment failure and worse patient outcomes [27–29], which is consistent with previous reports. Former studies proposed a percentage threshold method, and 30%–70% of SUVmax thresholds or 40%–80% SUVmax thresholds were used to generate MTV-based HI [14, 15, 26]. However, since the percentage threshold method strongly depends on tumor SUVmax, large differences exist between cancer lesions with high FDG uptake, which could not reflect tumor heterogeneity accurately.

Many studies indicated that ^18^F-FDG PET/CT image texture analysis was used to characterize the heterogeneity of tumor ^18^F-FDG uptake [30–32]. The texture analysis might predict the clinical outcome and treatment response of esophageal cancer [33], non-small-cell lung cancer [34], and locally advanced rectal cancer [35]. As a widely used method to evaluate tumor heterogeneity, texture analysis is not clinically available due to the inaccessible software in most imaging viewing workstations, the lack of established evaluation criteria, and the characteristics of time-consuming and complex [25]. Since it is challenging to obtain measurement results, it is difficult to evaluate texture analysis in clinical practice. By contrast, the calculation methods of PET metabolic heterogeneity parameters can be easily achieved on the commonly used workstation with high reproducibility.

Previous studies on the prediction of colorectal cancer have suggested that the N stage, CEA, MTV, TLG, TNM stage, and some other factors were related to the prognosis of colorectal cancer to some extent [22, 36–38]. Our study showed that recurrent patients in the radical treatment group had more tumor thrombus, higher tumor stage, and higher MTV and that regional lymph nodes status and tumor stage had more significant effects on prognosis than other variables, which is consistent with the findings of previous literature. Patients with larger active tumor volumes are more likely to have lymph vascular invasion [39], which relates to a poor prognosis. At present, TNM staging is the most commonly used prognostic model. Some pathological features associated with it, including lymph node metastasis or distant metastasis, can also partially reflect the prognosis accordingly. Notably, regional lymph node status was a risk factor in the univariate analysis but a protective factor in the multivariate analysis in the radical treatment group, which may be related to the positive correlation between regional lymph node status and tumor stage in the radical treatment group. In multivariate analysis, the tumor stage was a high multiplier risk factor, so the regional lymph node status associated with it may be a relative protective factor. In the analysis of OS in the palliative treatment group, all the parameters were not statistically significant; the reliability of these results is worth considering. It may be due to the small number of positive events in the sample and the subsequent errors or deviations in the statistical results. However, it can be solved by expanding the sample size or increasing the proportion of positive events by extending the follow-up time.

Some limitations should be mentioned. Firstly, the various treatment methods that the subjects received subsequently may affect the accuracy of the parameters. Therefore, the diversity of adjuvant therapy is a confounding factor that affects the outcome. Secondly, the observation time and the subject number are limited. Therefore, more extensive prospective studies involving many issues are required to confirm the findings.

## 5. Conclusions

In summary, the ^18^F-FDG PET/CT linear regression HI-2 value calculated based on MTV might serve as a predictor of prognosis for patients with colorectal cancer. Preoperative assessment of HI-2 might be a better indicator of intratumoral heterogeneity for prognostic inference of cancer patients due to its readily available nature. Meanwhile, the tumor stage and regional lymph nodes status could also predict PFS for patients with colorectal cancer.

## Figures and Tables

**Table 1:** Baseline clinicopathological and PET information of subjects.

Variables	Values
Gender	
** **Male	114 (60.6%)
** **Female	74 (39.4%)
Age, year	65 (55–75)
Location	
** **Right colon	59 (31.4%)
** **Left colon	64 (34.0%)
** **Rectum	65 (34.6%)
Pathological type	
** **Adenocarcinoma	159 (84.6%)
** **MAC/SRC	29 (15.4%)
Degree of differentiation.	
** **Low	44 (23.4%)
** **Median	137 (72.9%)
** **High	7 (3.7%)
Measured tumor length (cm)	4.90 ± 1.66
T Stage	
** **T_1-2_	37 (19.7%)
** **T_3-4_	151 (80.3%)
Regional lymph nodes	
** **N–	85 (45.2%)
** **N+	103 (54.8%)
Distant metastasis	
** **M–	129 (68.6%)
** **M+	59 (31.4%)
Nerve invasion	
** **Negative	148 (78.7%)
** **Positive	40 (21.3%)
Tumor thrombus	
** **Negative	135 (71.8%)
** **Positive	53 (28.2%)
Stage, AJCC	
** **I	25 (13.3%)
** **II	45 (23.9%)
** **III	59 (31.4%)
** **IV	59 (31.4%)
Type of surgery	
** **Radical surgery	129（68.6%）
** **Palliative surgery	59（31.4%）
CEA (ng/ml)	6.10 (2.77–21.84)
CA19-9 (U/ml)	17.17 (8.02–50.26)
Parameters of PET	
** **SUVmax	15.43 (11.98–20.91)
** **SUVpeak	10.67 (8.35–14.76)
** **SUVmean	8.91 (6.97–11.62)
** **MTV (ml)	12.17 (7.30–22.29)
** **TLG (g)	112.03 (62.88–214.86)
** **HI-1	0.23 (0.21–0.24)
** **HI-2	10.57 (6.05–19.21)

MAC, mucinous adenocarcinoma; SRC, signet ring cell carcinoma; CEA, carcinoembryonic antigen; CA19-9, carbohydrate antigen 19-9; SUV, standardized uptake value; MTV, metabolic tumor volume; TLG, total lesion glycolysis; HI, heterogeneity index.

**Table 2:** Comparison of clinicopathological and PET parameters between recurrence and no recurrence in 129 patients with curative radical surgery.

Variables	Recurrence (–)	Recurrence (+)	*P* value
Gender (male/female)	63/37	20/9	0.555
Age (median)	65	61	0.106
Location			
** **Right colon	33	8	
** **Left colon	28	9	
** **Rectum	39	12	0.856
Pathological type			
** **Adenocarcinoma	86	21	
** **MAC/SRC	14	8	0.087
Degree of differentiation			
** **Low	22	10	
** **Median	72	18	
** **High	6	1	0.368
Measured tumor length (cm) (mean ± SD)	4.86 ± 1.77	5.52 ± 1.70	0.075
T stage			
** **T_1-2_	29	5	
** **T_3-4_	71	24	0.206
Regional lymph nodes			
** **N–	37	10	
** **N+	63	19	0.804
Nerve invasion			
** **Negative	87	23	
** **Positive	13	6	0.304
Tumor thrombus			
** **Negative	87	16	
** **Positive	13	13	**＜0.001**
Stage, AJCC			
** **I	22	3	
** **II	41	4	
** **III	37	22	**0.001**
CEA (ng/ml) (median)	3.81	6.04	0.105
CA19-9 (U/ml) (median)	13.66	16.77	0.259
** **SUVmax (median)	15.37	14.40	0.348
** **SUVpeak (median)	10.67	10.81	0.552
** **SUVmean (median)	9.00	7.99	0.204
** **MTV (ml) (median)	10.28	12.87	**0.003**
** **TLG (g) (median)	97.55	135.16	0.073
** **HI-1 (median)	0.23	0.22	**0.032**
** **HI-2 (median)	8.29	17.74	**0.001**

MAC, mucinous adenocarcinoma; SRC, signet ring cell carcinoma; CEA, carcinoembryonic antigen; CA19-9, carbohydrate antigen 19-9; SUV, standardized uptake value; MTV, metabolic tumor volume; TLG, total lesion glycolysis; HI, heterogeneity index. Bold values, *P* < 0.05.

**Table 3:** Univariate and multivariate Cox regression analyses (radical treatment).

Variables	PFS						OS					
	Univariate analysis			Multivariate analysis			Univariate analysis			Multivariate analysis		
	HR	95% CI	*P* value	HR	95% CI	*P* value	HR	95% CI	*P* value	HR	95% CI	*P* value
Age	0.99	0.96–1.01	0.252				0.98	0.93–1.02	0.210			
Gender												
** **Male	1.00						1.00					
** **Female	0.76	0.36–1.59	0.458				0.25	0.03–2.06	0.199			
Tumor location												
** **Right colon	1.00						1.00					
** **Left colon	1.51	0.62–3.68	0.346				1.28	0.25–6.42	0.766			
** **Rectum	1.26	0.54–3.00	0.590				0.62	0.10–3.71	0.599			
Pathological types												
** **Adenocarcinoma	1.00						1.00					
** **MAC/SRC	2.05	0.95–4.42	0.068				1.63	0.33–8.11	0.551			
Differentiation												
** **LD	1.00						1.00					
** **MD/HD	0.77	0.37–1.63	0.498				0.97	0.20–4.81	0.971			
Tumor diameter	1.11	0.92–1.35	0.262				1.27	0.85–1.89	0.238			
T Stage												
** **T_1-2_	1.00						1.00					
** **T_3-4_	1.26	0.57–2.81	0.565				0.37	0.09–1.49	0.163			
Regional lymph nodes												
** **N–	1.00						1.00					
** **N+	2.54	1.26–5.12	**0.009**	0.16	0.04–0.57	**0.005**	1.56	0.39–6.24	0.533			
Nerve invasion												
** **Negative	1.00						1.00					
** **Positive	2.24	0.89–5.65	0.087				1.58	0.18–13.62	0.679			
Tumor thrombus												
** **Negative	1.00						1.00					
** **Positive	3.85	1.90–7.80	**<0.001**	1.48	0.65–3.36	0.351	3.19	0.76–13.43	0.115			
Stage, AJCC												
** **I-II	1.00						1.00					
** **III	3.67	1.74–7.75	**0.001**	20.65	4.81–88.62	**<0.001**	1.37	0.34–5.50	0.654			
CEA (ng/ml)												
** **Normal (≤5.00)	1.00						1.00					
** **Increased (>5.00)	1.94	1.00–3.90	0.061				4.50	0.90–22.45	0.067			
CA19-9 (U/ml)												
** **Normal (≤37.00)	1.00						1.00					
** **Increased (>37.00)	1.09	0.49–2.41	0.839				1.24	0.25–6.18	0.790			
SUVmax	0.97	0.92–1.02	0.256				1.00	0.91–1.11	0.938			
SUVmean	0.93	0.84–1.03	0.169				1.01	0.84–1.21	0.926			
SUVpeak	0.97	0.90–1.04	0.396				1.03	0.90–1.18	0.687			
MTV (ml)	1.02	1.01–1.04	**0.012**	0.98	0.96–1.01	0.279	1.03	1.00–1.06	**0.016**	0.96	0.92–1.01	0.122
TLG (g)	1.00	0.99–1.00	0.391				1.00	1.00–1.00	0.074			
HI-1												
** **≤0.23	1.00						1.00					
** **>0.23	0.52	0.25–1.11	0.089				0.42	0.08–2.07	0.284			
HI-2	1.07	1.04–1.10	**<0.001**	1.10	1.04–1.17	**0.001**	1.10	1.05–1.15	**<0.001**	1.16	1.07–1.26	**<0.001**

PFS, progression-free survival; OS, overall survival; MAC, mucinous adenocarcinoma; SRC, signet ring cell carcinoma; LD, low-differentiation; MD, middifferentiation; HD, High-differentiation; CEA, carcinoembryonic antigen; CA19-9, carbohydrate antigen 19-9; SUV, standardized uptake value; MTV, metabolic tumor volume; TLG, total lesion glycolysis; HI, heterogeneity index. Bold values, *P* < 0.05.

**Table 4:** Univariate and multivariate Cox regression analyses (palliative treatment).

Variables	PFS						OS					
	Univariate analysis			Multivariate analysis			Univariate analysis			Multivariate analysis		
	HR	95% CI	*P* value	HR	95% CI	*P* value	HR	95% CI	*P* value	HR	95% CI	*P* value
Age	0.99	0.97–1.01	0.347				1.02	0.97–1.06	0.502			
Gender												
** **Male	1.00						1.00					
** **Female	1.51	0.80–2.83	0.200				1.35	0.46–3.90	0.586			
Tumor location												
** **Right colon	1.00						1.00					
** **Left colon	0.88	0.43–1.79	0.724				0.45	0.15–1.39	0.167			
** **Rectum	0.67	0.28–1.63	0.379				0.18	0.02–1.42	0.102			
Pathological types												
** **Adenocarcinoma	1.00						1.00					
** **MAC/SRC	0.80	0.24–2.61	0.71				1.76	0.38–8.08	0.470			
Differentiation												
** **LD	1.00						1.00					
** **MD/HD	1.17	0.54–2.55	0.687				0.61	0.20–1.82	0.372			
Tumor diameter	1.13	0.91–1.39	0.280				1.38	0.99–1.93	0.059			
T stage												
** **T_1-2_	1.00						1.00					
** **T_3-4_	2.94	0.40–21.60	0.289				-	-	-			
Regional lymph nodes												
** **N−	1.00						1.00					
** **N+	2.05	0.86–4.91	0.106				1.66	0.37–7.44	0.511			
Nerve invasion												
** **Negative	1.00						1.00					
** **Positive	1.13	0.60–2.14	0.696				1.32	0.46–3.82	0.607			
Tumor thrombus												
** **Negative	1.00						1.00					
** **Positive	1.80	0.96–3.36	0.067				2.55	0.85–7.65	0.095			
CEA (ng/ml)												
** **Normal (≤5.00)	1.00						1.00					
** **Increased (>5.00)	1.47	0.61–3.52	0.388				1.83	0.40–8.40	0.436			
CA19-9 (U/ml)												
** **Normal (≤37.00)	1.00						1.00					
** **Increased (>37.00)	1.44	0.77–2.70	0.251				2.50	0.83–7.47	0.102			
SUVmax	1.04	1.00–1.08	0.063				1.02	0.94–1.09	0.683			
SUVmean	1.07	1.00–1.14	0.062				1.02	0.89–1.15	0.825			
SUVpeak	1.07	1.01–1.13	**0.025**	1.04	0.97–1.11	0.318	1.02	0.92–1.13	0.710			
MTV (ml)	1.01	1.00–1.03	0.075				1.00	0.99–1.03	0.530			
TLG (g)	1.00	0.99–1.00	**0.012**	1.00	1.00–1.00	0.712	1.00	0.99–1.00	0.502			
HI-1												
** **≤0.23	1.00						1.00					
** **>0.23	0.57	0.29–1.12	0.103				0.66	0.20–2.13	0.485			
HI-2	1.04	1.02–1.07	**0.001**	1.03	1.01–1.06	**0.020**	1.03	1.00–1.06	0.061			

PFS, progression-free survival; OS, overall survival; MAC, mucinous adenocarcinoma; SRC, signet ring cell carcinoma; LD, low-differentiation; MD, middifferentiation; HD, high-differentiation; CEA, carcinoembryonic antigen; CA19-9, carbohydrate antigen 19-9; SUV, standardized uptake value; MTV, metabolic tumor volume; TLG, total lesion glycolysis; HI, heterogeneity index. Bold values, *P* < 0.05.

## Data Availability

The dataset generated or analyzed in this study is included with this paper and can be made available from the corresponding author upon reasonable request.

## Supplementary Materials

Supplementary Materials Appendix 1: differences in ^18^F-FDG PET/CT traditional and heterogeneity parameters between groups with different pathological characteristics.

## References

[1] Shi L., Guo H., Zheng Z., Liu J., Jiang Y., Su Y. (2020). Laparoscopic surgery versus open surgery for colorectal cancer: impacts on natural killer cells, cancer control. *Journal of the Moffitt Cancer Center*.

[2] Cook A. D., Single R., McCahill L. E. (2005). Surgical resection of primary tumors in patients who present with stage IV colorectal cancer: an analysis of surveillance, epidemiology, and end results data, 1988 to 2000. *Annals of Surgical Oncology*.

[3] Kuipers E. J., Grady W. M., Lieberman D. (2015). Colorectal cancer. *Nature Reviews. Disease Primers*.

[4] National Health Commission Of The People’s Republic Of China (2020). National guidelines for diagnosis and treatment of colorectal cancer 2020 in China (English version). *Chinese journal of cancer research = Chung-kuo yen cheng yen chiu*.

[5] Nors J., Henriksen T. V., Gotschalck K. A. (2020). IMPROVE-IT2: implementing noninvasive circulating tumor DNA analysis to optimize the operative and postoperative treatment for patients with colorectal cancer - intervention trial 2. Study protocol. *Acta Oncologica*.

[6] Chiu K. W. H., Lam K.-O., An H. (2018). Long-term outcomes and recurrence pattern of 18F-FDG PET-CT complete metabolic response in the first-line treatment of metastatic colorectal cancer: a lesion-based and patient-based analysis. *BMC Cancer*.

[7] Zhao K., Wang C., Shi F. (2021). Combined prognostic value of the SUVmax derived from FDG-PET and the lymphocyte-monocyte ratio in patients with stage IIIB-IV non-small cell lung cancer receiving chemotherapy. *BMC Cancer*.

[8] Wang W., Liu G., Hu P. (2020). Imaging characteristics and prognostic values of hepatic epithelioid hemangioendothelioma on 18F-FDG PET/CT. *Clinical and Experimental Medicine*.

[9] Kitajima K., Miyoshi Y., Yamano T., Odawara S., Higuchi T., Yamakado K. (2018). Prognostic value of FDG-PET and DWI in breast cancer. *Annals of Nuclear Medicine*.

[10] Flatin B. T. B., Vedeld H. M., Pinto R. (2021). Multiregional assessment of CIMP in primary colorectal cancers: phenotype concordance but marker variability. *International Journal of Cancer*.

[11] Liu G., Yin H., Cheng X. (2021). Intra-tumor metabolic heterogeneity of gastric cancer on 18F-FDG PETCT indicates patient survival outcomes. *Clinical and Experimental Medicine*.

[12] Zhao Y., Liu C., Zhang Y., Gong C., Li Y., Xie Y. (2018). Prognostic value of tumor heterogeneity on 18F-FDG PET/CT in HR+HER2- metastatic breast cancer patients receiving 500 mg fulvestrant: a retrospective study. *Scientific Reports*.

[13] Lee M., Lee H., Cheon G. J. (2017). Prognostic value of preoperative intratumoral FDG uptake heterogeneity in patients with epithelial ovarian cancer. *European Radiology*.

[14] Kimura M., Kato I., Ishibashi K. (2019). The prognostic significance of intratumoral heterogeneity of 18F-FDG uptake in patients with oral cavity squamous cell carcinoma. *European Journal of Radiology*.

[15] Kwon S. H., Yoon J.-K., An Y.-S. (2014). Prognostic significance of the intratumoral heterogeneity of 18 F-FDG uptake in oral cavity cancer. *Journal of Surgical Oncology*.

[16] Gerlinger M., Rowan A. J., Horswell S. (2012). Intratumor heterogeneity and branched evolution revealed by multiregion sequencing. *New England Journal of Medicine*.

[17] Asselin M.-C., O’Connor J. P. B., Boellaard R., Thacker N. A., Jackson A. (2012). Quantifying heterogeneity in human tumours using MRI and PET. *European Journal of Cancer*.

[18] Shi D., Cai G., Peng J. (2015). The preoperative SUVmax for 18F-FDG uptake predicts survival in patients with colorectal cancer. *BMC Cancer*.

[19] Shangguan C., Gan G., Zhang J. (2018). Cancer-associated fibroblasts enhance tumor 18F-FDG uptake and contribute to the intratumor heterogeneity of PET-CT. *Theranostics*.

[20] Kocael A., Vatankulu B., Şimşek O. (2016). Comparison of 18F-fluorodeoxyglucose PET/CT findings with vascular endothelial growth factors and receptors in colorectal cancer. *Tumor Biology*.

[21] Jo H. J., Kim S.-J., Lee H. Y., Kim I. J. (2014). Prediction of survival and cancer recurrence using metabolic volumetric parameters measured by 18F-FDG PET/CT in patients with surgically resected rectal cancer. *Clinical Nuclear Medicine*.

[22] Ogawa S., Itabashi M., Kondo C., Momose M., Sakai S., Kameoka S. (2015). Prognostic value of total lesion glycolysis measured by 18F-FDG-PET/CT in patients with colorectal cancer. *Anticancer Research*.

[23] Kim Y.-i., Paeng J. C., Cheon G. J. (2016). Prediction of posttransplantation recurrence of hepatocellular carcinoma using metabolic and volumetric indices of 18F-FDG PET/CT. *Journal of Nuclear Medicine*.

[24] Chung H. H., Kang S. Y., Ha S. (2016). Prognostic value of preoperative intratumoral FDG uptake heterogeneity in early stage uterine cervical cancer. *Journal of gynecologic oncology*.

[25] Kim Y.-i., Kim Y. J., Paeng J. C. (2017). Heterogeneity index evaluated by slope of linear regression on 18F-FDG PET/CT as a prognostic marker for predicting tumor recurrence in pancreatic ductal adenocarcinoma. *European Journal of Nuclear Medicine and Molecular Imaging*.

[26] Kim T. H., Yoon J.-K., Kang D. K. (2015). Correlation between F-18 fluorodeoxyglucose positron emission tomography metabolic parameters and dynamic contrast-enhanced MRI-derived perfusion data in patients with invasive ductal breast carcinoma. *Annals of Surgical Oncology*.

[27] Razzak M. (2014). New molecular classification of gastric adenocarcinoma proposed by the Cancer Genome Atlas. *Nature Reviews Clinical Oncology*.

[28] Giganti F., Antunes S., Salerno A. (2017). Gastric cancer: texture analysis from multidetector computed tomography as a potential preoperative prognostic biomarker. *European Radiology*.

[29] Li M., Ke J., Wang Q. (2015). Upregulation of ROCK2 in gastric cancer cell promotes tumor cell proliferation, metastasis and invasion. *Clinical and Experimental Medicine*.

[30] Cook G. J. R., O’Brien M. E., Siddique M. (2015). Non-small cell lung cancer treated with erlotinib: heterogeneity of18F-FDG uptake at PET-association with treatment response and prognosis. *Radiology*.

[31] Lee H. S., Oh J. S., Park Y. S., Jang S. J., Choi I. S., Ryu J.-S. (2016). Differentiating the grades of thymic epithelial tumor malignancy using textural features of intratumoral heterogeneity via 18F-FDG PET/CT. *Annals of Nuclear Medicine*.

[32] Hyun S. H., Kim H. S., Choi S. H. (2016). Intratumoral heterogeneity of 18F-FDG uptake predicts survival in patients with pancreatic ductal adenocarcinoma. *European Journal of Nuclear Medicine and Molecular Imaging*.

[33] Nakajo M., Jinguji M., Nakabeppu Y. (2017). Texture analysis of 18F-FDG PET/CT to predict tumour response and prognosis of patients with esophageal cancer treated by chemoradiotherapy. *European Journal of Nuclear Medicine and Molecular Imaging*.

[34] Cook G. J. R., Yip C., Siddique M. (2013). Are pretreatment 18F-FDG PET tumor textural features in non-small cell lung cancer associated with response and survival after chemoradiotherapy?. *Journal of Nuclear Medicine*.

[35] Bundschuh R. A., Dinges J., Neumann L. (2014). Textural parameters of tumor heterogeneity in 18F-FDG PET/CT for therapy response assessment and prognosis in patients with locally advanced rectal cancer. *Journal of Nuclear Medicine*.

[36] Xu J., Li Y., Hu S., Lu L., Gao Z., Yuan H. (2019). The significant value of predicting prognosis in patients with colorectal cancer using 18F-FDG PET metabolic parameters of primary tumors and hematological parameters. *Annals of Nuclear Medicine*.

[37] Li Destri G., Rubino A. S., Latino R. (2015). Preoperative carcinoembryonic antigen and prognosis of colorectal cancer. An independent prognostic factor still reliable. *International Surgery*.

[38] Shia J., Klimstra D. S., Bagci P., Basturk O., Adsay N. V. (2012). TNM staging of colorectal carcinoma: issues and caveats. *Seminars in Diagnostic Pathology*.

[39] Chen X. L., Chen G. W., Pu H. (2019). DWI and T2-weighted MRI volumetry in resectable rectal cancer: correlation with lymphovascular invasion and lymph node metastases. *AJR. American journal of roentgenology*.

