# Supplementary material for: Prognostic Value of Intratumor Metabolic Heterogeneity Parameters on 18F-FDG PET/CT for Patients with Colorectal Cancer
Source: Contrast Media Mol Imaging. 2022 Jan 30;2022:2586245. doi: 10.1155/2022/2586245 (PMC8818395; doi:10.1155/2022/2586245)
Supplement: Supplementary Materials — Appendix 1: differences in 18F-FDG PET/CT traditional and heterogeneity parameters between groups with different pathological characteristics. [file 2586245.f1.doc]

| **Supplement Table 1. Differences in ^18^F-FDG PET/CT parameters between groups** | | | | | | | | | | | | | |
| --- | --- | --- | --- | --- | --- | --- | --- | --- | --- | --- | --- | --- | --- |
| **Variables (median)** | **T_1-2_** | **T_3-4_** | ***P* value** | **N^-^** | **N^+^** | ***P* value** | **M^-^** | **M^+^** | ***P* value** | **TT^-^** | **TT^+^** | ***P* value** |  |
| **SUVmax** | 14.45 | 15.47 | 0.490 | 15.27 | 15.47 | 0.502 | 15.24 | 15.96 | 0.672 | 15.47 | 15.39 | 0.589 |  |
| **SUVmean** | 8.67 | 9.00 | 0.534 | 9.00 | 8.79 | 0.412 | 8.83 | 9.23 | 0.647 | 9.00 | 8.67 | 0.536 |  |
| **SUVpeak** | 10.18 | 10.73 | 0.255 | 10.97 | 10.56 | 0.395 | 10.72 | 10.57 | 0.971 | 10.72 | 10.56 | 0.845 |  |
| **MTV** | 8.17 | 12.71 | **0.028** | 10.48 | 12.71 | 0.188 | 11.47 | 13.12 | 0.066 | 11.47 | 14.61 | **0.015** |  |
| **TLG** | 86.94 | 123.47 | **0.046** | 99.60 | 118.72 | 0.523 | 112.35 | 111.71 | 0.330 | 97.79 | 133.71 | 0.067 |  |
| **HI-1** | 0.23 | 0.23 | 0.084 | 0.23 | 0.23 | 0.202 | 0.23 | 0.23 | 0.932 | 0.23 | 0.22 | 0.094 |  |
| **HI-2** | 6.03 | 10.89 | **0.002** | 9.08 | 11.55 | 0.089 | 9.74 | 11.06 | **0.046** | 9.74 | 14.36 | **0.024** |  |
| **Variables (median)** | **NI^-^** | **NI^+^** | ***P* value** | **Adenocarcinoma** | **MAC/**  **SRC** | ***P* value** | **LD** | **MD/**  **HD** | ***P* value** | **Right Colon** | **Left Colon** | ***P* value** |  |
| **SUVmax** | 15.24 | 16.37 | 0.426 | 15.39 | 16.62 | 0.623 | 14.30 | 15.81 | 0.342 | 16.12 | 14.40 | 0.545 |  |
| **SUVmean** | 8.75 | 9.48 | 0.431 | 8.83 | 9.00 | 0.478 | 7.84 | 9.15 | 0.215 | 9.12 | 8.23 | 0.506 |  |
| **SUVpeak** | 10.57 | 11.68 | 0.350 | 10.72 | 10.57 | 0.640 | 10.37 | 10.85 | 0.577 | 11.55 | 10.37 | 0.404 |  |
| **MTV** | 11.64 | 15.02 | 0.199 | 11.14 | 19.89 | **0.011** | 16.18 | 11.18 | **0.008** | 16.18 | 10.85 | **0.022** |  |
| **TLG** | 100.49 | 134.13 | 0.160 | 99.60 | 161.27 | 0.053 | 142.31 | 98.70 | 0.053 | 160.60 | 93.63 | **0.029** |  |
| **HI-1** | 0.23 | 0.23 | 0.525 | 0.23 | 0.22 | **0.045** | 0.23 | 0.23 | 0.300 | 0.22 | 0.23 | 0.263 |  |
| **HI-2** | 9.78 | 13.08 | **0.045** | 9.58 | 17.00 | **0.011** | 17.01 | 9.70 | **0.006** | 17.00 | 9.20 | **0.018** |  |

Abbrevious：SUV, standardized uptake value; MTV, metabolic tumor volume; TLG, total lesion glycolysis; HI, heterogeneity index; N, regional lymph node metastasis;

M, distant metastasis; TT, tumor thrombus; NI, nerve invasion; MAC, Mucinous adenocarcinoma; SRC, Signet ring cell carcinoma;LD, low-differentiated; MD, Mid-

differentiated; HD, High-differentiated.
